# A case report of pituitary neuroendocrine tumor manifesting as severe conjunctival chemosis

**DOI:** 10.1186/s12886-023-03224-5

**Published:** 2023-11-22

**Authors:** Shun Yamamuro, Atsuo Yoshino, Takuma Nishide, Hiroshi Negishi, Takahiro Kumagawa

**Affiliations:** Department of Neurological Surgery, Nihon University School of Medicine, 30-1 Oyaguchi Kami-Cho, Itabashi-Ku, Tokyo, 173-8610 Japan

**Keywords:** Conjunctival chemosis, Pituitary neuroendocrine tumor, Transsphenoidal surgery, Cavernous sinus, Oculomotor nerve palsy

## Abstract

**Background:**

Conjunctival chemosis (CC) is an extremely rare symptom of pituitary neuroendocrine tumor (PitNET). We report an extremely rare case of PitNET manifesting as severe CC.

**Case presentation:**

A 48-year-old male was admitted to our hospital with severe CC, proptosis, and ptosis of the right eye. Magnetic resonance imaging demonstrated the tumor mass invading the cavernous sinus (CS) with cystic lesion. The patient underwent emergent endoscopic transsphenoidal surgery, and the pathological diagnosis was PitNET. CC of the right eye remarkably improved after the surgery. Glucocorticoid therapy was performed for right oculomotor nerve palsy, which rapidly improved. The postoperative course was uneventful and the patient was discharged from our hospital without hormone replacement.

**Conclusions:**

CC caused by CS invasion of PitNET can be cured by early surgical treatment. Therefore, PitNET is important to consider in the differential diagnosis of CC.

## Background

Pituitary neuroendocrine tumor (PitNET) which has invaded cavernous sinus (CS) often causes compression of the oculomotor nerve or other cranial nerves, and manifests as oculomotor nerve palsy or other symptoms [1–5]. Such symptoms are particularly associated with rapid growth of the tumor due to complications caused by pituitary apoplexy [1–5]. Conjunctival chemosis (CC) is a common symptom of carotid cavernous fistula (CCF), but is rarely caused by PitNET or pituitary apoplexy. We report a very rare case of PitNET manifesting as severe CC.

## Case presentation

**Fig. 1:**
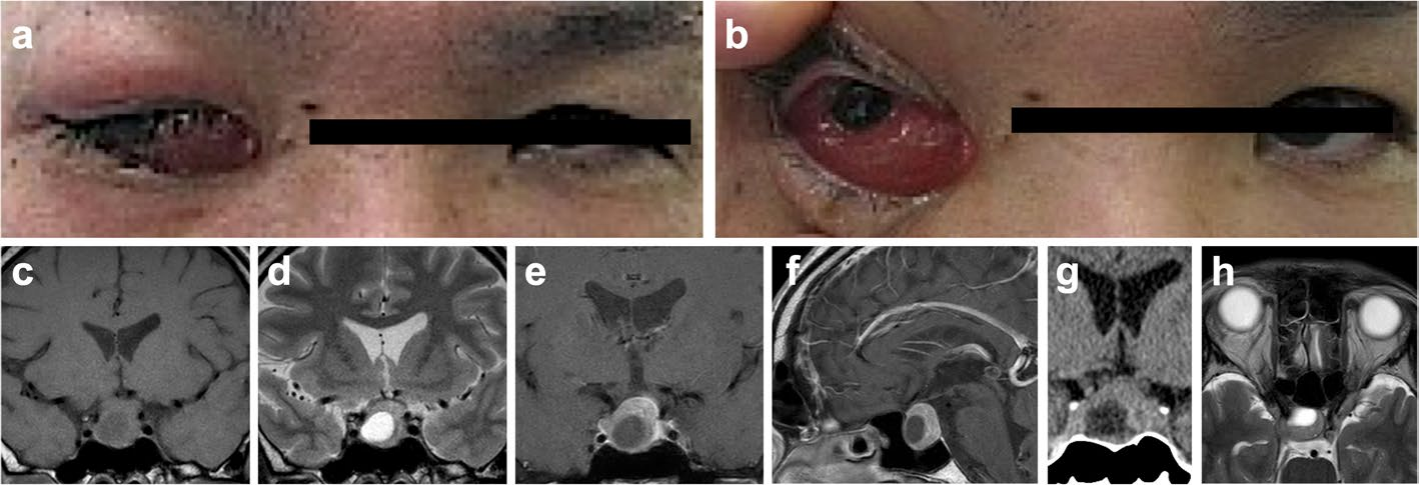
**a**, **b** Preoperative photographs of the patient’s face demonstrating marked right eye conjunctival chemosis (**a**: opening by self, **b**: opening manually). **c**–**f** Findings of initial magnetic resonance images (**c**: coronal T1-weighted imaging, **d**: coronal T2-weighted imaging, **e**: coronal gadolinium-enhanced T1-weighted imaging, **f**: sagittal gadolinium-enhanced T1-weighted imaging). **g** Findings of initial coronal computed tomography scan. **h** Findings of initial axial T2-weighted magnetic resonance image focusing around the orbits

A 48-year-old male was admitted to our hospital with suffering from severe CC of the right eye (Fig. 1a, b), ocular movement disorder, and ptosis. The patient had no particularly medical, family, and psycho-social history. The patient complained of dryness and hyperemia in his right eye for 2 weeks prior to admission, and CC developed 1 week prior to admission. He was referred to our hospital, since these symptoms did not improve despite administration of eye drops. His consciousness level was clear and neurological examinations revealed no abnormalities except for the symptoms of the right eye. Laboratory evaluations including hormonal tests did not detect any abnormalities. The basal levels of anterior and posterior pituitary hormones were within the normal range with no increase or decrease, and no symptoms of diabetes insipidus or electrolyte abnormalities were observed. Magnetic resonance (MR) imaging revealed a 2-cm sellar mass lesion with cyst formation (Fig. 1c–f). The mass extended to the suprasellar region and invaded the right CS, and appeared as iso-intensity on T1-weighted and T2-weighted MR imaging, with heterogeneous enhancement after contrast medium administration. The cystic lesion appeared as low intensity on T1-weighted MR imaging, high intensity on T2-weighted MR imaging, without enhancement after contrast medium administration. MR angiography did not reveal CCF, aneurysms, or other abnormal findings. Computed tomography (CT) provided no findings suggesting either acute or old hemorrhage in the mass (Fig. 1g). Axial T2-weighted MR imaging demonstrated dilation of the superior ophthalmic vein (SOV) (Fig. 1h), so we thought the pituitary tumor had infiltrated the CS resulting in increased pressure within the CS, causing perfusion disorder of the SOV. The patient underwent emergent endoscopic transsphenoidal surgery, and the pituitary tumor was almost totally resected. The mass was soft and easy to curette. The fluid inside the cyst did not demonstrate high pressure, and was almost clear liquid with a slight yellowish tinge. No typical evidence of old hemorrhage suggesting pituitary apoplexy was recognized intraoperatively. Pathological examination demonstrated a typical adenoma pattern of diffuse sheets consisting of eosinophilic PitNET cells, and these findings were consistent with PitNET. Immunohistochemically, tumor cells were very weakly positive for growth hormone, negative for adrenocorticotropic hormone, thyroid stimulating hormone, and follicle stimulating hormone. The MIB-1 labeling index was < 1%.

**Fig. 2:**
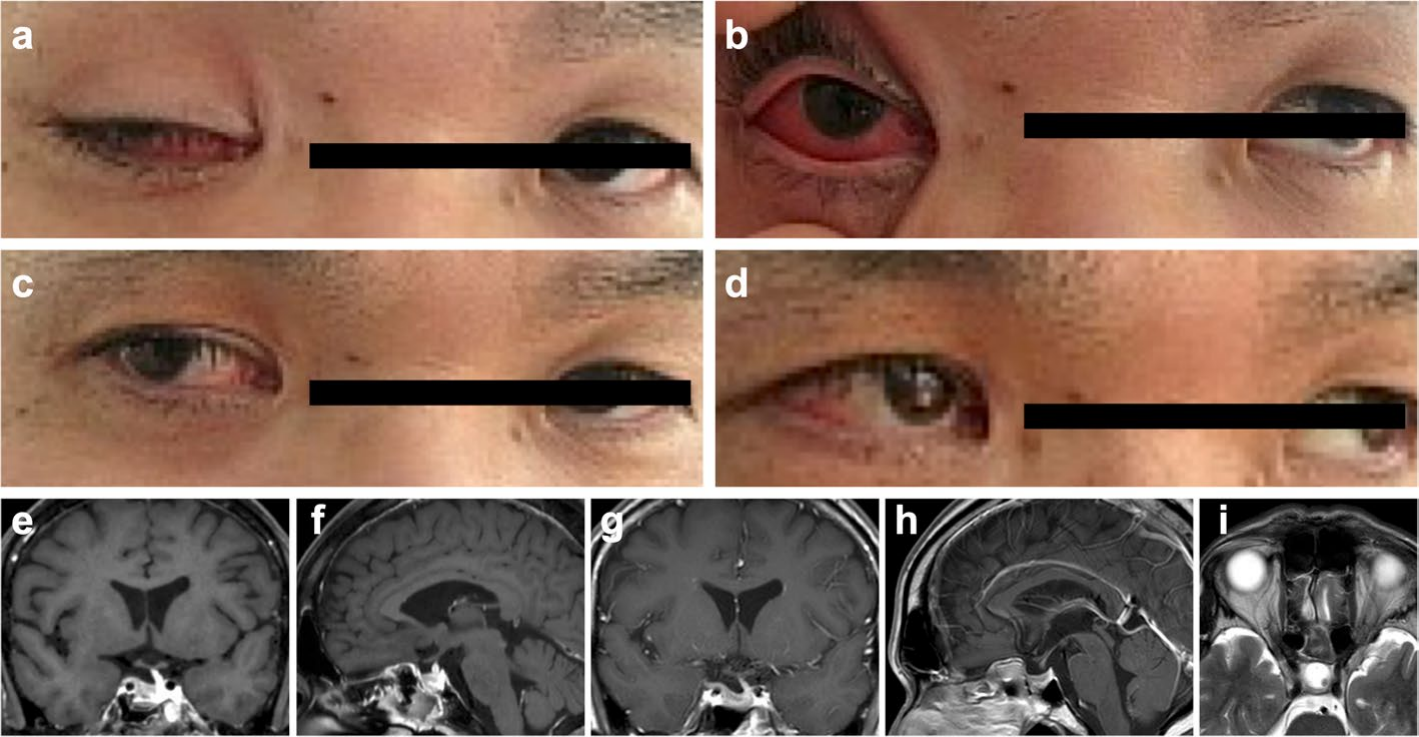
**a**, **b** Postoperative photographs of the patient’s face (**a**: opening by self, **b**: opening manually). **c**, **d** Photographs of the patient’s face after glucocorticoid treatment (**c**: eight gaze, **d**: left gaze). **e**, **f** Findings of magnetic resonance images immediately after the surgery (**e**: coronal gadolinium-enhanced T1-weighted imaging, **f**: sagittal gadolinium-enhanced T1-weighted imaging). **g**, **h** Findings of magnetic resonance images 3 months after the surgery (**g**: coronal gadolinium-enhanced T1-weighted imaging, **h**: sagittal gadolinium-enhanced T1-weighted imaging). **i** Findings of axial T2-weighted magnetic resonance image 3 months after the surgery focusing around the orbits

CC of the right eye had remarkably improved 1 week after the surgery (Fig. 2a, b). As the CC improved, the symptoms of right oculomotor nerve palsy, especially impaired adduction of the eye, became more prominent. Therefore, prednisolone was prescribed starting at 60 mg/day and finishing by tapering every 4 days (total glucocorticoid treatment period was 28 days). The oculomotor nerve palsies were particularly improved after the glucocorticoid treatment (Fig. 2c, d). The patient’s condition continued to improve and he was discharged from our hospital without hormone replacement therapy (Fig. 2e, f). There were no hormonal deficits in the evaluation immediately after surgery and 3 months after surgery, and no symptoms of diabetes insipidus were observed. MR imaging 3 months after the surgery demonstrated no recurrence of the tumor and dilation of the SOV had disappeared (Fig. 2g–i).

## Discussion and conclusion

CC is not recognized as a symptom of PitNET, so is rarely considered when a patient complains of CC. The present case is apparently the only reported instance of PitNET manifesting as severe CC. Our patient with CC caused by PitNET invading the CS was cured by early surgical treatment. Therefore, PitNET is important to consider in the differential diagnosis of CC. We think that the CC in this case was caused by perfusion disorder in the SOV via the CS infiltrated by PitNET. SOV dilation is characteristically observed in CS disease, such as dural CCF [6, 7]. However, SOV dilation is rarely caused by PitNET and has never been reported. Furthermore, many cases of PitNET are known with much greater CS invasion than the present case, but cases of PitNET with CC was previously unreported.

Preoperatively, we thought that the CC in our case was caused by pituitary apoplexy. Pituitary apoplexy may cause increased pressure in the CS and SOV due to a rapid increase in tumor volume. In addition, inflammatory cytokines, which can induce vasodilation or cytokine storm, may have been released by pituitary apoplexy and entered SOV, resulting in dilation of the conjunctival vessels [8]. However, the characteristic symptoms and laboratory findings of pituitary apoplexy were absent. The PitNET of the present case was associated with a cystic lesion, but MR imaging and CT did not suggest acute hemorrhage (Fig. 1c, d, g). In addition, the characteristic symptoms of pituitary apoplexy, such as headache, fatigue, and panhypopituitarism, were absent [9–12]. Basal hormone levels were normal both before and after surgery, and no hormone replacement was necessary. Most cases of PitNET complicated by oculomotor nerve palsy manifest as pituitary apoplexy [1–5], but the diagnosis becomes even more difficult in the absence of pituitary apoplexy. Pituitary apoplexy has recently been suggested to represent primarily hemorrhagic infarction [12, 13], but pituitary apoplexy with only infarction without hemorrhage has also been reported [9]. MR imaging demonstrated only infarction without overt hemorrhage in 30% of cases of pituitary apoplexy [9]. However, the cystic lesion in the present case appeared as low intensity on diffusion-weighted MR imaging (data not shown), suggesting no acute infarction had occurred. Furthermore, intraoperative findings and pathological examinations also failed to identify findings suggestive of infarction.

Another possibility is that the venous blood outflow from PitNET, which normally flows into CS, was interrupted and instead flowed into the SOV for some reason. However, severe ocular symptoms are usually more likely to occur with high blood flow shunts through direct CCF of the internal carotid artery and CS, and are less likely with low blood flow shunts through indirect fistulas [14]. In particular, CC is more likely to occur during high flow shunt due to direct fistulas from the internal carotid artery to CS [14]. Therefore, the mechanism of CC in the present case is difficult to explain.

As a point to reflection, we should have performed dynamic vascular study, such as digital subtraction angiography, 3D-CT angiography / venography or MR venography, before the surgery. It would be considered that some sort of triggered event such as intra-tumoral hemorrhage should be required to develop CC. If the CC occurred via reasons other than intra-tumoral hemorrhage, preexisting anatomical condition, such as undeveloped venous return from the cavernous sinus, must have existed. Therefore, we should have performed dynamic vascular study and completely ruled out other factors for developing CC. Furthermore, we should have evaluated existence of the occult arteriovenous shunt, such as caused via ruptured cavernous sinus aneurysm, by dynamic vascular study before surgery. If the surgery performed without adequate preoperative examination, surgeon might encounter uncontrollable bleeding from the cavernous sinus.

In the present case, oculomotor nerve palsy, which we had not been able to effectively evaluate preoperatively due to the severe CC, manifested after the improvement of CC. The possibility of this symptom resulting from side effects and complications due to the surgery cannot be excluded. However, the oculomotor nerve palsy markedly improved after immediate glucocorticoid therapy. Glucocorticoid therapy is reportedly effective for both idiopathic or traumatic oculomotor nerve palsy [15]. On the other hand, the supportive evidence is unclear. Glucocorticoid therapy was also reported to have no direct therapeutic effect on oculomotor nerve palsy, and oculomotor nerve palsy simply recovers through natural progression and/or rehabilitation [16]. In the present case, the oculomotor nerve palsy may have spontaneously resolved without glucocorticoid therapy. Conversely, the symptoms may not have improved without glucocorticoid therapy. Therefore, therapeutic evidence for oculomotor nerve palsy could be established in the future.

Future experience with similar cases will help to elucidate the pathophysiology of this complicated CC presentation. Delays in diagnosis and surgical treatment are believed to have significant negative impacts on the patient's functional prognosis in such cases. Therefore, intracranial diseases such as PitNET should be suspected in patients presenting with eye symptoms for which no clear causative disease is identified.

## Data Availability

The data of the case are available from the corresponding author on reasonable request.

## Abbreviations

CC: Conjunctival chemosis
CCF: Carotid-cavernous fistula
CS: Cavernous sinus
MR: Magnetic resonance
PitNET: Pituitary neuroendocrine tumor
SOV: Superior ophthalmic vein
